# Clinical and laboratory characteristics of complex febrile seizures in the acute phase: a case-series study in Japan

**DOI:** 10.1186/s12883-023-03051-7

**Published:** 2023-01-18

**Authors:** Tsukasa Tanaka, Hiroshi Yamaguchi, Yusuke Ishida, Kazumi Tomioka, Masahiro Nishiyama, Daisaku Toyoshima, Azusa Maruyama, Hiroki Takeda, Hiroshi Kurosawa, Ryojiro Tanaka, Kandai Nozu, Hiroaki Nagase

**Affiliations:** 1 Department of Pediatrics, Kobe University Graduate School of Medicine, 7-5-2, Kusunoki-Cho, Chuo-Ku, Hyogo 650-0017 Kobe, Japan; 2 Department of Neurology, Hyogo Prefectural Kobe Children’s Hospital, Kobe, Japan; 3 Department of Pediatric Critical Care Medicine, Hyogo Prefectural Kobe Children’s Hospital, Kobe, Japan; 4 Department of Emergency and General Medicine, Hyogo Prefectural Kobe Children’s Hospital, Kobe, Japan

**Keywords:** Children, Complex febrile seizures, Encephalitis, Encephalopathy, Clinical characteristics, Laboratory characteristics

## Abstract

**Background:**

Patients with complex febrile seizures (CFS) often display abnormal laboratory results, unexpectedly prolonged seizures, and/or altered consciousness after admission. However, no standardized values have been established for the clinical and laboratory characteristics of CFS in the acute phase, making the management of CFS challenging. This study aimed to determine the clinical and laboratory characteristics of children with CFS during the acute phase. In particular, the duration of impaired consciousness and the detailed distribution of blood test values were focused.

**Methods:**

We retrospectively reviewed medical records of a consecutive pediatric cohort aged 6–60 months who were diagnosed with CFS and admitted to Kobe Children’s Hospital between October 2002 and March 2017. During the study period, 486 seizure episodes with confirmed CFS were initially reviewed, with 317 seizure episodes included in the analysis. Detailed clinical and laboratory characteristics were summarized.

**Results:**

Among 317 seizure episodes (296 children with CFS), 302 required two or fewer anticonvulsants to be terminated. In 296 episodes showing convulsive seizures, median seizure duration was 30.5 min. The median time from onset to consciousness recovery was 175 min. Impaired consciousness lasting > 6, 8, and 12 h was observed in 13.9%, 7.6%, and 1.9% patients with CFS, respectively. Additionally, the distribution of aspartate aminotransferase, lactate dehydrogenase, creatinine, and glucose were clarified with 3, 10, 50, 90, and 97 percentile values.

**Conclusion:**

This study detailed the clinical and laboratory findings of acute-phase CFS using the data of the largest 15-year consecutive cohort of children with CFS. These results provide important information for appropriate acute management of CFS.

## Background

Febrile seizures (FS) are the most common seizure disorder in childhood, affecting 2–5% of children in the United States and 3.4–9.3% in Japan [1–5]. As defined by the American Academy of Pediatrics, FS occur in the absence of intracranial infection, metabolic disturbance, or a history of afebrile seizures, and are classified as simple or complex [6].

Simple FS (SFS) are isolated, brief, and generalized seizures [7]. Conversely, complex FS (CFS) are defined as those with focal onset, prolonged duration (greater than 10–15 min), or those that occur more than once within the same febrile illness [7, 8]. Approximately 35% of FS cases are classified as CFS [9].

Children with SFS usually do not require hospitalization. However, patients with CFS often have prolonged seizures and/or impaired consciousness or have an accompanying serious infection or infection whose etiology cannot be rapidly determined. Therefore, hospitalization is recommended for observation. In fact, several international guidelines also recommend routine admission for observation of all patients presenting with CFS [10, 11]; in our hospital, we also hospitalize patients with CFS.

The acute management of CFS depends on the seizure status and level of consciousness of the patient upon arrival at the hospital and on admission [4]. Recent literature suggests that routine lumbar puncture and urgent neuroimaging are not essential for all CFS cases [12, 13]. Therefore, physicians often perform routine laboratory tests and close observation during the acute management of CFS.

However, no standard values have been established for the clinical and laboratory characteristics of CFS in the acute phase. Therefore, appropriate acute management of CFS is difficult in the absence of reliable information when patients with CFS display abnormalities in laboratory results, prolonged seizures, or persistent altered consciousness at an early stage.

Accordingly, this study aimed to describe the basic epidemiological data regarding clinical and laboratory characteristics among children with CFS during the acute phase.

## Methods

### Patients

This study is a retrospective analysis based on a database review. All methods were performed in accordance with the relevant guidelines and regulations. We created a database of patients aged 1 month to 15 years who were admitted for fever and seizures to Kobe Children’s Hospital, a tertiary referral hospital where children with CFS are routinely admitted. We assessed consciousness using the Glasgow Coma Scale (GCS) in children recovering from seizures at least at hourly intervals. In the original cohort, we retrospectively reviewed the consecutive case records of children aged 6–60 months who were diagnosed with CFS between October 2002 and March 2017. Patients with incomplete medical data and a history of neurological disorders were excluded. Patients who needed intensive care, such as the use of continuous anticonvulsants or treatment with targeted temperature management, were also excluded because consciousness could not be evaluated.

### Definitions of seizures

FS are defined as seizures accompanied by fever (temperature ≥ 38.0 °C) without central nervous system infection that occur in infants or children aged 6–60 months. Patients with a prior history of epilepsy were excluded from the FS category. CFS are defined as FS with one or more of the following three features: focal manifestations, prolonged (≥15 min) duration, and recurrence within 24 h according to the new Japanese guidelines for the management of FS [14]. Patients with temperatures of ≥38.0 °C within 24 h before or after seizure onset were treated as febrile cases and were included in this study, even if their temperature was lower than 38.0 °C upon admission.

### Clinical and laboratory data

The following information was collected from patient records: age, sex, history of FS, body temperature at admission, presence of convulsive seizures, classification of CFS, total number and type of anticonvulsants used, duration of convulsive seizures, duration of impaired consciousness, and blood examination results after seizure onset.

The timing of seizure onset was determined based on the interviews with parents or observers, emergency transport records, referrals, and/or medical records. In cases with multiple seizures, the data of the final seizure that determined hospitalization were chosen and extracted.

In children who presented with convulsive seizures, the duration of the seizures was calculated as the time from the onset to the end of convulsive movement. When sequential convulsive seizures without full recovery of consciousness were observed, the duration of the seizures was determined from the beginning of the first convulsion to the end of the last convulsion. The duration of impaired consciousness was calculated as the time between seizure onset and a GCS score of 15 was confirmed by history or observation.

Blood tests included the following: white blood cell count, hemoglobin, platelet count, albumin, aspartate aminotransferase (AST), alanine aminotransferase, lactate dehydrogenase (LDH), creatine kinase, creatinine (Cr), sodium, glucose (Glu), C-reactive protein, pH, base excess, lactate, and prothrombin time. When multiple values were available for blood data, the first values recorded after the onset of seizures, which determined hospitalization, were selected for analysis.

## Results

**Fig. 1:**
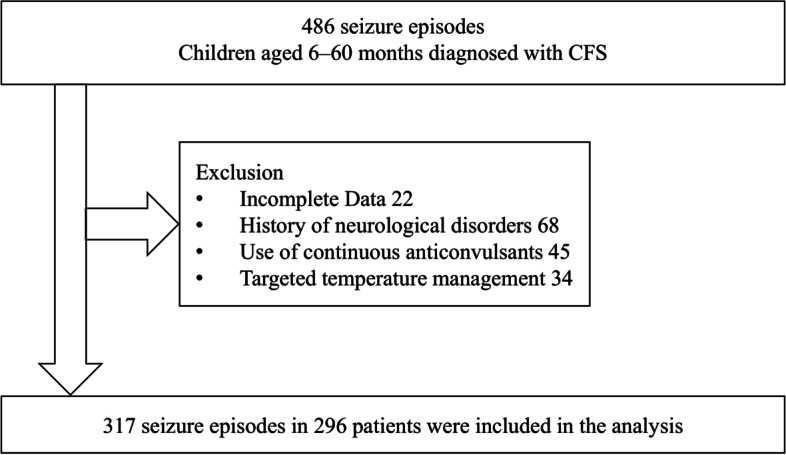
Flowchart of study enrollment. CFS, complex febrile seizures

During the study period, 486 episodes of seizures with confirmed CFS were recorded. We excluded the following number of seizure episodes: 22 with incomplete data, 68 with a history of neurological disorders, 45 with continuous anticonvulsant administration, and 34 with targeted temperature management. The remaining 317 seizure episodes that occurred in 296 children were included in the analysis (Fig. 1). During the study period, 278 children, 17 children, and one child experienced CFS once, twice, and five times, respectively.

**Table 1:** Clinical characteristics of pediatric patients who were hospitalized for CFS (*n *= 317)

Characteristics	Value	
Age, months	22.5	(6.2–59.1)
Male sex	174	(54.9)
History of FS	92	(29.0)
Temperature at admission, °C	38.9 ± 1.0	
Convulsive seizures	296	(93.4)
Classification of CFS		
Focal seizures	196	(61.8)
Prolonged convulsive seizures ≥ 15 min	186	(58.7)
Repetitive seizures	112	(35.3)
Total number of anticonvulsants used		
0	106	(33.4)
1	140	(44.2)
2	56	(17.7)
3	14	(4.4)
4	1	(0.3)
Details of anticonvulsants used		
Diazepam iv	131	(41.3)
Diazepam sp	74	(23.3)
Midazolam iv	66	(20.8)
Midazolam buc	2	(0.6)
Fosphenytoin iv	23	(7.3)
Phenytoin iv	1	(0.3)
Phenobarbital iv	4	(1.3)
Thiamylal iv	1	(0.3)
Carbamazepine per os	8	(2.5)

Data are presented as the median (range), mean ± standard deviation, or number (%)

*buc* buccal administration, *CFS* complex febrile seizures, *FS* febrile seizures, *iv* intravenous administration, *sp* suppository administration

**Fig. 2:**
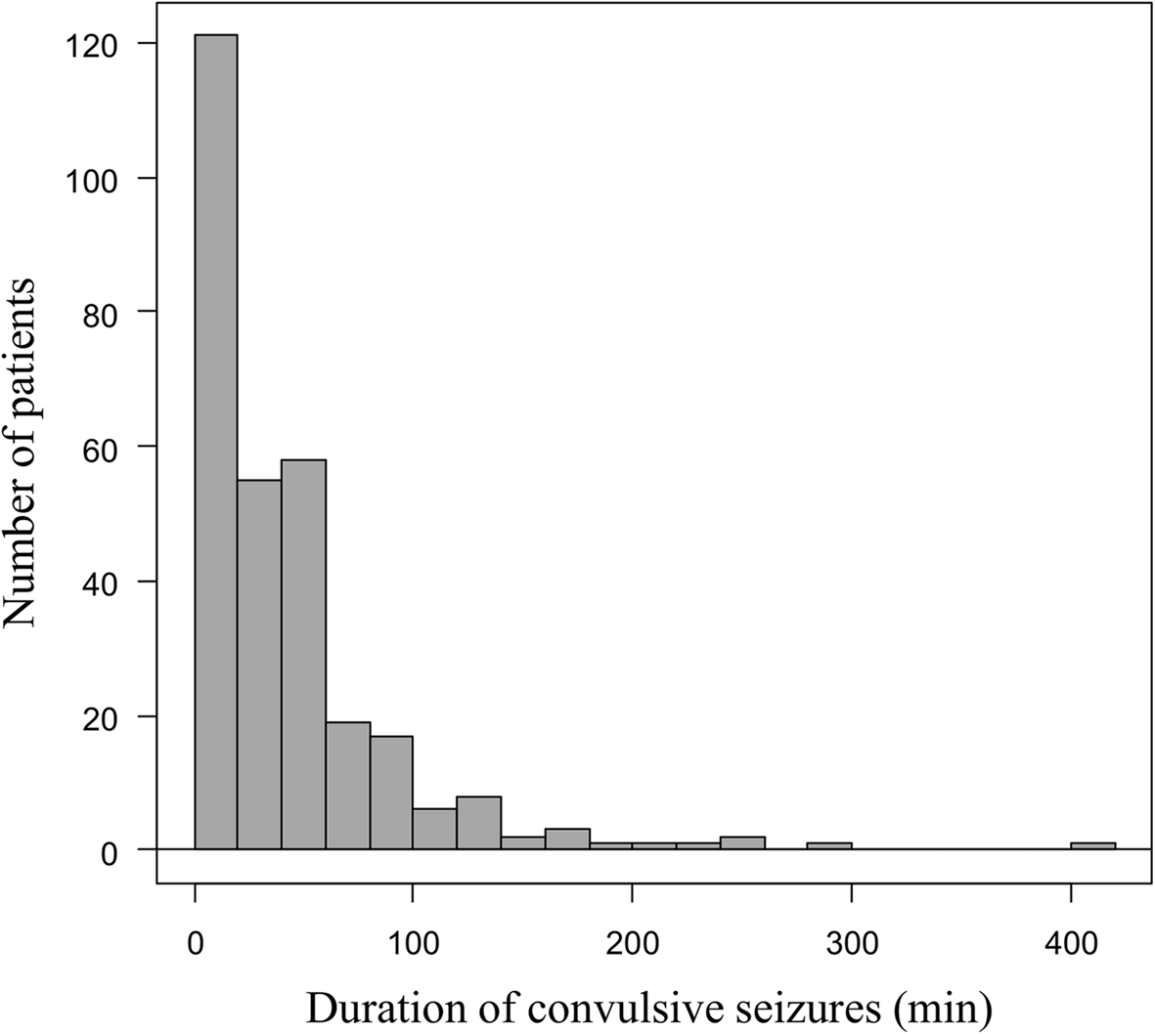
Distribution of convulsive seizure duration in patients with complex febrile seizures. The horizontal axis shows the duration of convulsive seizures, separated by 20 min intervals. The vertical axis shows the number of patients

The patient’s clinical characteristics are summarized in Table 1. Convulsive seizures were observed in 277 patients, with 296 seizure episodes (93.4%). As for anticonvulsants, 302 (95.3%) seizure episodes required two or fewer anticonvulsants to terminate the seizures. Intravenous or suppository diazepam was administered for 205 seizure episodes (64.7%) and intravenous midazolam was administered for 66 seizure episodes (20.8%). Intravenous fosphenytoin was used for 23 seizure episodes (7.3%). Few children were treated with intravenous phenytoin, phenobarbital, thiamylal, buccal midazolam, or oral carbamazepine, all of which in total were administered for only 16 seizure episodes (5.0%). Figure 2 shows the distribution of the duration of convulsive seizures, with a median duration of 30.5 (range: 1–410) min. Of the 296 convulsive seizure episodes, 148 episodes (50%) terminated within 30 min, 234 (79.1%) terminated within 1 h, and 276 (93.2%) terminated within 2 h.

**Fig. 3:**
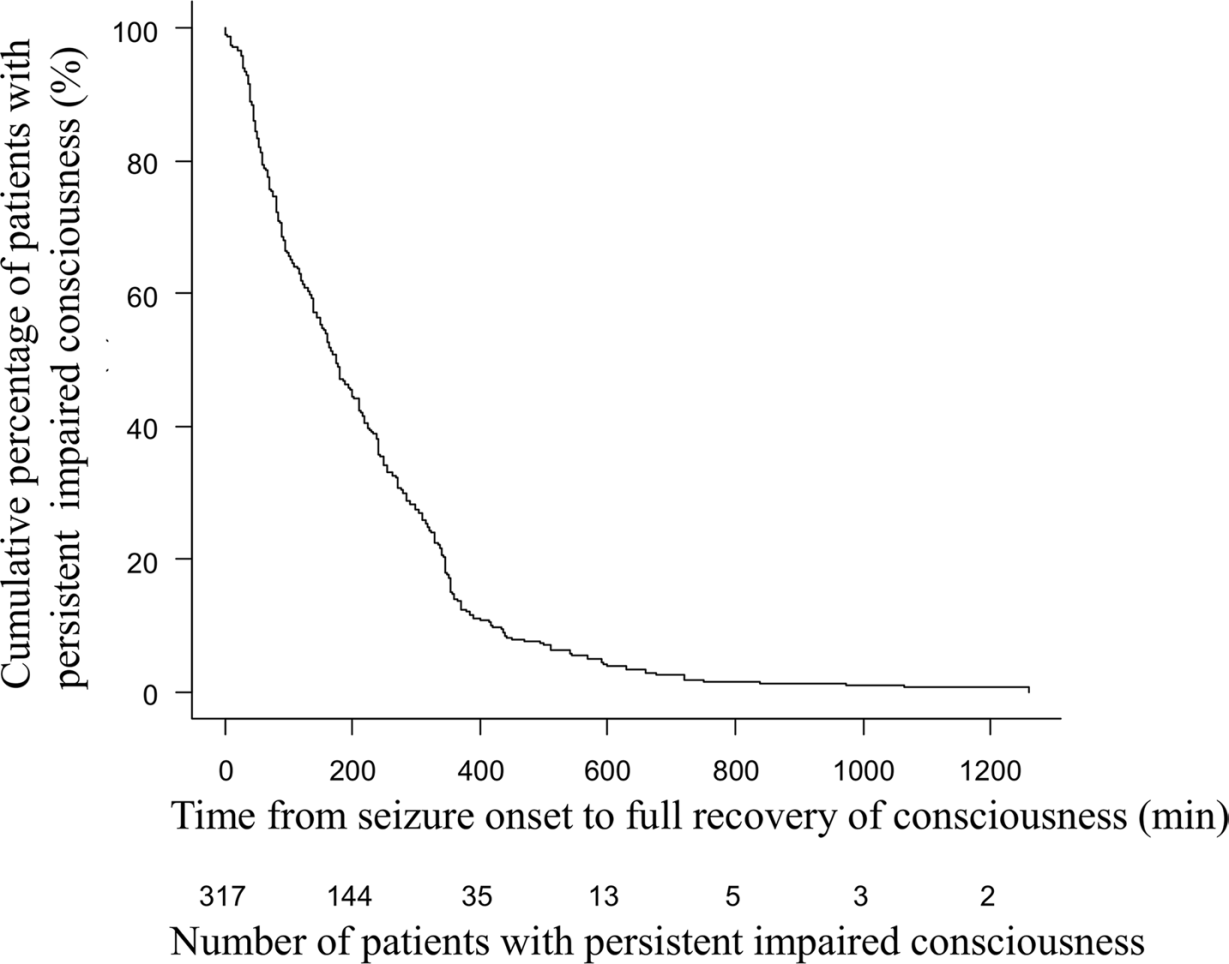
Relationship between time from seizure onset to full recovery of consciousness and cumulative percentage of patients with persistent impaired consciousness in patients with complex febrile seizures. Vertical axis shows the cumulative percentage of patients with persistent impaired consciousness. Horizontal axis shows time from seizure onset to full recovery of consciousness and the number of patients with persistent impaired consciousness

Figure 3 shows the relationship between the time from seizure onset and the cumulative percentage of patients with persistent impaired consciousness, demonstrating that the median time from seizure onset to full recovery of consciousness was 175 (range: 1–1260) min, and impaired consciousness lasting > 6, 8, and 12 h was observed in 13.9%, 7.6%, and 1.9% of the patients, respectively.

Table 2 shows the laboratory characteristics of patients with CFS. Our findings clarified the percentile distribution of multiple laboratory parameters as well as the mean and standard deviation. This information indicates that abnormal laboratory values are common in patients with CFS and reveals the actual percentage and extent of deviations from normal values.

**Table 2:** Laboratory characteristics of patients with CFS (*n *= 317)

Parameter		Mean	SD	3^rd^ centile	10^th^ centile	50^th^ centile	90^th^ centile	97^th^ centile
WBC (*n *= 314)	(/µL)	12,336	6339	4200	5900	10,900	20,370	27,215
Hb (*n *= 314)	(g/dL)	11.8	0.9	10.0	10.6	11.9	12.9	13.4
PLT (*n *= 314)	(10^4^/µl)	29.2	10.1	15.1	18.6	27.8	40.6	50.8
ALB (*n *= 67)	(mg/dL)	4.3	0.4	3.7	3.8	4.3	4.7	4.9
AST (*n *= 314)	(IU/L)	42	46	25	27	36	54	74
ALT (*n *= 314)	(IU/L)	20	37	9	11	15	26	43
LDH (*n *= 307)	(IU/L)	307	79	204	237	293	391	468
CK (*n *= 294)	(IU/L)	129	58	57	66	118	200	273
Cre (n = 311)	(mg/dL)	0.27	0.08	0.17	0.20	0.26	0.34	0.40
Na (*n *= 313)	(mEq/L)	134.6	3.0	129	131	135	138	140
CRP (*n *= 307)	(mg/dL)	1.22	1.96	0.03	0.06	0.52	2.86	7.28
Glu (*n* = 310)	(mg/dL)	141	49	80	91	130	207	266
pH (*n *= 280)		7.295	0.462	6.898	7.103	7.362	7.443	7.471
BE (*n *= 280)		-4.4	3.7	-12.9	-9.9	-3.85	-0.9	0.9
Lac (*n *= 272)	(mmol/L)	2.4	1.3	0.8	1.2	2.1	3.9	5.4
PT (*n *= 182)	(%)	80	17	48	61	79.5	100	114

*ALB* albumin, *ALT* alanine aminotransferase, *AST* aspartate aminotransferase, *BE* base excess, *CFS* complex febrile seizures, *CK* creatine kinase, *Cre* creatinine, *CRP* C-reactive protein, *Glu* glucose, *Hb* hemoglobin, *Lac* lactate, *LDH* lactate dehydrogenase, *Na* sodium, *PLT* platelet, *PT* prothrombin time, *SD* standard deviation, *WBC* white blood cell

## Discussion

In the present study, we described the clinical and laboratory characteristics of CFS that required hospitalization. Although large studies on clinical features of febrile seizures have recently been published [15, 16], to the best of our knowledge, this is the first report to describe the detailed characteristics of acute-phase CFS.

Our study showed that the median duration of convulsive seizures was 30.5 min and the median time from seizure onset to full recovery of consciousness was 175 min in patients with CFS. This differs from the findings of previous reports on seizure duration and recovery time. Okumura et al. [17] examined the clinical features of prolonged unconsciousness and delirious behavior in children with FS and showed that the duration of seizures was < 5 min in 90.2% and the duration of unconsciousness was < 30 min in 93% of the seizures, leading to the conclusion that prolonged unconsciousness is rare in children with FS. In prior studies of epileptic seizures conducted by Allen et al. [18] and McKenny-Fick et al. [19], the median duration and recovery time of FS were 2.5 and 18 min, respectively. The discrepancy between our study and previous studies can be explained by the difference in the study participants. Patients with SFS were not included in our study, while the previous studies included patients with SFS. In addition, the hospital setting may have influenced this difference. While the previous reports mentioned above were conducted in hospitals providing secondary-level pediatric care, our hospital is a tertiary referral hospital for children. Therefore, there may be a tendency for more severe cases to be referred to our hospital instead of to other institutes.

In a previous study on febrile status epilepticus (FSE), which was defined as FS without full recovery lasting ≥ 30 min, Shinnar et al. [20] reported the consequences of 119 cases of FSE, showing that seizures lasted for a median of 68 min, 24% of the seizures lasted > 2 h, and 87% of the seizures did not stop spontaneously and were treated with benzodiazepines. Our study demonstrated that convulsive seizures of CFS lasted a median of 30.5 min and 6.8% of the convulsive seizures lasted > 2 h, and two-thirds of the seizures required anticonvulsants. In addition, half of the participants had FSE. In our study, the severity of seizures was milder than that reported by Shinnar et al. [20] because our study also included patients who had seizures lasting < 30 min. However, it is particularly worth noting that half of the patients with CFS had FSE. Berg et al. [9] reported that CFS and FSE accounted for approximately 35% and 5% of all FS, respectively. Therefore, our patients with CFS had more FSE than previously reported. We may have encountered only some of the more severe cases of CFS.

The most notable aspect of our study is its detailed focus on the duration of impaired consciousness and the distribution of blood test results. Prolonged impaired consciousness and abnormal laboratory values are expected to be more common in patients with CFS and FSE than in those with SFS. However, almost all previous reports on CFS and/or FSE, including that of Shinnar et al. [20], did not mention the details of the duration of impaired consciousness or blood test values. Therefore, these points could be considered the greatest novelty of this study.

The definitive diagnosis of CFS in the acute phase is sometimes challenging because patients with both CFS and acute encephalitis/encephalopathy (AEE) exhibit impaired consciousness with or without seizures as an initial manifestation. There is a well-established consensus on the clinical case definition and diagnostic methods for encephalitis [21]. However, in some cases of AEE, there may be no significant cerebrospinal fluid pleocytosis or no demonstrable neuroimaging abnormalities [21]. Therefore, complete differentiation between CFS and AEE at an early stage can sometimes be impossible in practice and often confounds clinicians, especially when symptoms during the acute phase are severe. Our previous report on the risk factors related to poor outcomes in patients with fever and seizures showed that refractory status epilepticus, consciousness disturbance or hemiplegia at 6 h from onset, or AST > 90 IU/L within 6 h of onset were associated with poor outcomes, leading to the final diagnosis of AEE [22, 23]. In addition, several reports have suggested that prolonged impaired consciousness and abnormal laboratory findings such as elevation of AST, LDH, Glu, and/or Cr might predict poor outcomes or AEE in children with seizures [24–30]. These abnormalities are sometimes observed in CFS, but their details in CFS have remained unclear. Our study is the first report to describe this information of acute-phase CFS, showing that impaired consciousness lasting > 6, 8, and 12 h was observed in 13.9%, 7.6%, and 1.9% of CFS cases, and 97 percentile values of AST, LDH, Cre, and Glu were 74 IU/L, 468 IU/L, 0.40 mg/dL, and 266 mg/dL, respectively. This information may be useful in that data outside of these ranges should alert an alternative diagnosis other than CFS at an early stage. Therefore, our findings may provide important information for appropriate acute management of CFS.

The present study has several limitations. First, this study was based on data from a single tertiary care institution; hence, our hospital is more likely to encounter more severe cases than other institutions. Second, since severe cases requiring intensive care were excluded, only mild cases may have been extracted, resulting in some selection bias. Third, even though we presented detailed data on the recovery time of consciousness, we did not identify independent factors that affected it. Previous reports have shown that the use of antiepileptic drugs significantly increased recovery time [18, 19]. In our report, two-thirds of patients were treated with one or more anticonvulsants. Therefore, drug administration may have resulted in a longer time to complete recovery of consciousness. However, factors other than drug type that affect recovery time need to be considered, such as the actual drug dose administered and the time taken from onset to the start of treatment. The present study design did not provide sufficient information on the drug dose and time from onset to treatment initiation, and thus it was not possible to examine factors that independently affected recovery time. Finally, this study only described the detailed characteristics of acute-phase CFS and did not compare the findings with those of patients with related disorders such as SFS or AEE. Therefore, the findings of this study do not allow us to differentiate CFS from these conditions. To confirm the clinical validity of the findings of this study as reliable information for the differential diagnosis of CFS, comparative studies with SFS and AEE are needed.

## Conclusion

The present study describes the detailed clinical and laboratory findings of CFS in an acute setting by reviewing the largest 15-year consecutive data of children with CFS. These findings may provide important information for appropriate acute management of CFS.

## Data Availability

The datasets generated and/or analyzed during the current study are available from the corresponding author on reasonable request.

## Abbreviations

FS: Febrile seizures
SFS: Simple febrile seizures
CFS: Complex febrile seizures
GCS: Glasgow Coma Scale
AST: Aspartate aminotransferase
LDH: Lactate dehydrogenase
Cr: Creatinine
Glu: Glucose
FSE: Febrile status epilepticus
AEE: Acute encephalitis/encephalopathy
